# Radiographic Assessment of Transverse Tarsometatarsal Instability Complicated by Metatarsus Adductus in Hallux Valgus Patients

**DOI:** 10.3390/life14060718

**Published:** 2024-06-01

**Authors:** Shun-Ping Wang, Cheng-Min Shih, Yu-Hsien Wu, Yuan-Shao Chen

**Affiliations:** 1 Department of Orthopaedic Surgery, Taichung Veterans General Hospital, Taichung 407219, Taiwan; wsp0120@vghtc.gov.tw (S.-P.W.); stargate@vghtc.gov.tw (C.-M.S.); inty246@vghtc.gov.tw (Y.-H.W.); 2 Department of Post-Baccalaureate Medicine, College of Medicine, National Chung Hsing University, Taichung 402202, Taiwan; 3 Department of Physical Therapy, HungKuang University, Taichung 433304, Taiwan

**Keywords:** metatarsus adductus, hallux valgus, M1-2 distance, TMT instability, MAA, HVA, IMA

## Abstract

Objective evaluations of transverse tarsometatarsal (TMT) hypermobility/instability are lacking. This study aims to radiographically explore the relationship between transverse TMT instability and metatarsus adductus (MA) in hallux valgus (HV). This study retrospectively analyzed 207 feet with varying degrees of HV, employing the distance between the first and second metatarsals (M1-2 distance) to assess transverse TMT instability of the first ray. Participants were categorized into MA and non-MA groups. It was found that the M1-2 distance significantly increased with the hallux valgus angle (HVA) and metatarsus adductus angle (MAA), demonstrating significant differences between the MA and non-MA groups. The measurement of M1-2 distance showed high reliability, and its cutoff value was determined to be 4.05 mm. Additionally, the results suggest that the widening of the M1-2 distance may be a predisposing factor for MA in HV patients, highlighting its role in the pathogenesis of this foot condition. These findings highlight the need for a comprehensive assessment of TMT instability on both the axial and sagittal planes for the surgical planning of HV, particularly when complicated by a large MAA. Based on these insights, reoriented first-TMT arthrodesis might be recommended for HV with significant MA to address potential multiplanar instability.

## 1. Introduction

Metatarsus adductus (MA) is a prevalent congenital foot deformity, occurring in approximately 1 to 2 per 1000 live births [1]. It is characterized as a uniplanar transverse-plane deformity at the Lisfranc joint, leading to metatarsal adduction [2]. Studies indicate that the prevalence of MA among patients with hallux valgus (HV) varies between 21.6% and 29.6% [3,4]. HV, a common forefoot deformity, is identified by a lateral deviation of the hallux, accompanied by an increased hallux valgus angle (HVA) and intermetatarsal angle (IMA), and other radiographic abnormalities [5,6]. Significant correlations between MA and HV have been observed in both adults and adolescents [7,8,9], with MA patients being approximately 3.5 times more likely to develop HV [8].

Several radiological methods have been developed to assess the metatarsus adductus angle (MAA) for diagnosing MA [10]. Among these, three radiographic measurements—Sgarlato’s MAA (MAA4), modified Sgarlato’s MAA (MAA5), and modified Engel’s MAA—demonstrate relatively high inter-observer reliability and are recommended for the diagnosis of MA [10,11]. It has been noted that MA can adversely affect surgical outcomes, leading to higher mean post-operative HVA, a higher revision rate, and an increased recurrence rate in HV populations [4,12,13]. In cases of HV with MA, a reduced first IMA must be considered in the planning of surgical approaches and techniques [14,15]. Furthermore, surgical corrections involving re-stabilization of the first tarsometatarsal (TMT-1) joint using a modified Lapidus procedure have been reported to yield superior outcomes compared to metatarsal osteotomies, particularly in terms of recurrence rates in HV patients with MA [13]. These findings imply the potential instability of the TMT-1 joint in HV patients with MA.

First-ray hypermobility and instability, involving the sagittal and axial planes at the TMT-1 joint, are recognized as contributing factors to the development of HV and its recurrence post-operatively [16,17]. Additionally, instability of the TMT-1 joint in HV patients predisposes them to axial malalignment [18]. Studies have demonstrated a correlation between an enlarged IMA and increased maximum dorsiflexion of the first ray, in terms of TMT-1 sagittal instability, during dynamic gait evaluations in HV patients [19]. Recent assessments using weight-bearing computed tomography (CT) have identified multiplanar instability of the TMT-1 joint in HV [20]. However, while most research on first-ray instability has focused on the sagittal plane [18,21,22], investigations into transverse instability specific to HV patients remain relatively limited [20,23].

At present, there is no agreed-upon method for objectively evaluating transverse instability in HV using radiography. In this research, the distance between the first and second metatarsal bases (M1-2 distance) on axial foot radiographs is defined as a measure of transverse TMT instability. This approach is similar to assessments used in diagnosing ligamentous Lisfranc injuries that involve traumatic separation of the intermetatarsal bases [24]. To date, the relationship between transverse instability of the TMT joint in HV and MA remains unclear. The M1-2 distance was assessed in both normal individuals and HV patients to establish a cutoff value, clarifying the extent of transverse instability in the TMT-1 joint and its association with HA and MAA. These findings aim to provide clinicians with deeper insights into MA in HV and serve as a scientific basis for refining surgical strategies in cases of HV with pronounced MA.

This study has two objectives. The first is to evaluate alterations in the M1-2 distance in patients with HV and to establish a normative value for the cohort. The second goal is to investigate whether the M1-2 distance increases in the MA population. The hypothesis is that an increased M1-2 distance correlates with the presence of MA in patients with HV.

## 2. Materials and Methods

### 2.1. Patient Recruitment

This study received approval from the Institutional Review Board (IRB) of our institution (No. CE14084). The retrospective enrollment of patients, both with and without HV, was conducted using medical records and images from August 2013 to December 2022. All participants recruited in the study had weight-bearing dorsoplantar (DP) and lateral view foot radiographs available. HV was diagnosed when the HVA exceeded 15° on the DP view of standing foot radiographs. Only symptomatic HV patients who had undergone further surgical treatment were enrolled. One hundred and one patients were identified with HV (HV group) and forty-six without (control group), totaling 147 patients and 294 feet, all of whom were included for further analysis.

**Figure 1:**
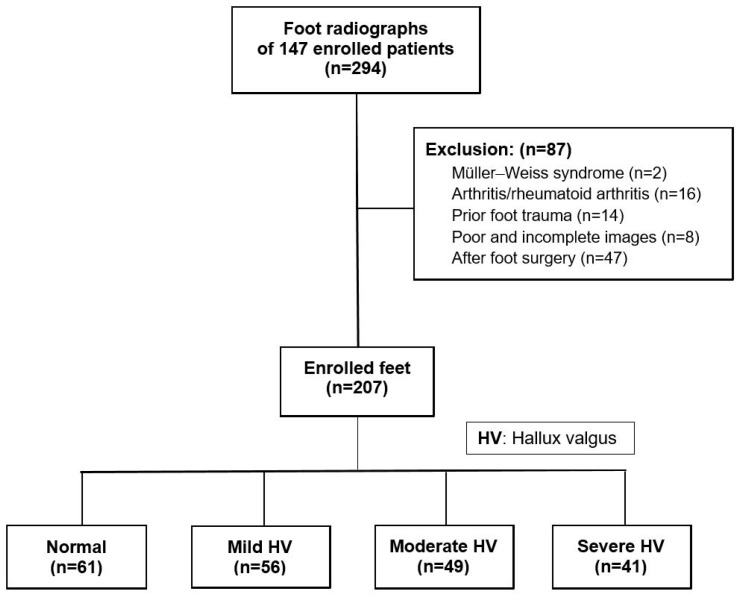
Flowchart of enrolled cases.

The exclusion criteria were as follows: (1) individuals under the age of 18, (2) incomplete or poor-quality images, (3) significant foot arthritis or deformity, (4) history of prior trauma or infection, and (5) previous surgical intervention on the ankle or foot. Ultimately, after the exclusion of 87 feet, measurements of the radiographic parameters were performed on 207 feet (Figure 1).

### 2.2. Radiographic Measurements

**Figure 2:**
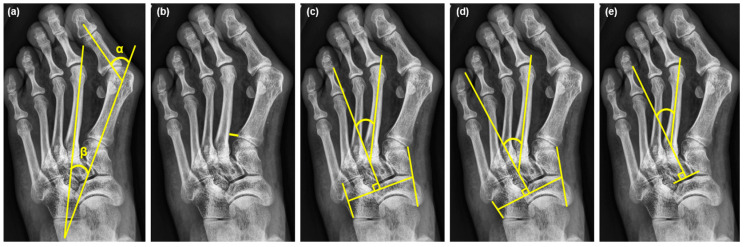
The radiographic measurements. (**a**) **α**: hallux valgus angle (HVA), **β**: intermetatarsal angle (IMA); (**b**) the distance between the first and second metatarsal bases (M1-2 distance); (**c**) Sgarlato’s MAA (MAA4); (**d**) modified Sgarlato’s MAA (MAA5); (**e**) modified Engel’s MAA. These weight-bearing foot radiographs of a 62-year-old female hallux valgus patient with metatarsus adductus provided the following measurements: HVA: 51°, IMA: 13°, MAA4: 28.05°, MMA5: 34.28°, modified Engel’s MAA: 33.07°, and M1-2 distance: 4.2 mm.

In this study, standard radiologic criteria were employed, and all radiographic parameters were measured using the built-in software of the Picture Archiving and Communications System (PACS) with ultraquery technology (Taiwan Electronic Data Processing, Sindian City, Taiwan). Measurements were taken from the weight-bearing DP and lateral-view radiographs of the foot. The radiological parameters assessed included HVA, IMA, M1-2 distance, and three different measurements of MAA: MAA4, MAA5, and modified Engel’s MAA [11]. The definitions of the radiographic measurements are as follows. HVA: the angle between the longitudinal axis of the first metatarsal and the first proximal phalanx [25]. IMA: the angle between the first and second metatarsals [25]. M1-2 distance: the shortest distance between the proximal articular cortexes of the first and second metatarsal bases [24]. MAA4: the angle between the longitudinal axis of the second metatarsal and the longitudinal axis of the lesser tarsus, using the fourth metatarso-cuboid joint as a reference [11]. MAA5: the angle between the longitudinal axis of the second metatarsal and the longitudinal axis of the lesser tarsus, using the fifth metatarso-cuboid joint as a reference [11]. Modified Engel’s MAA: the angle between the longitudinal axes of the middle cuneiform and the second metatarsal (Figure 2) [11].

### 2.3. The Severity of HV and Cutoff Value of MAA

The participants in this study were categorized into four subgroups based on the severity of HV, using the HVA as a criterion. The classifications were defined as follows: normal (<15°), mild (15–30°), moderate (30–40°), and severe (>40°) [26]. Additionally, the diagnostic cutoff values for MA were established from the MMA measurements—MAA4 at 14°, MAA5 at 20°, and modified Engel’s MAA at 24°. These thresholds were adopted based on previously reported literature, and they facilitated the confirmation of MA diagnoses among the participants [11].

### 2.4. The Intraclass Correlation Coefficient (ICC) Analysis and Determination of the Cutoff Value of the M1-2 Distance

In this investigation, radiographs from 40 patients were analyzed, with the patients being randomly selected and evenly distributed into four subgroups representing different severities of HV (10 cases per subgroup). Two authors (one orthopedic surgeon and one specialist in the foot and ankle) independently measured the target parameters on these radiographs at two separate time points to perform an ICC analysis. Both the intra- and inter-observer reliability were assessed to verify the reproducibility of the measurement of the M1-2 distance. Regarding the establishment of the cutoff value for the M1-2 distance, only cases classified as normal (61 feet) and mild HV (56 feet) were included to determine this value, in order to minimize bias potentially introduced by more severe HV or higher HVA values. The cutoff value was set at two standard deviations (SDs) above the mean M1-2 distance [27], aiming to establish a reliable threshold for differentiating between normal and pathological conditions.

### 2.5. Statistical Analysis

Continuous variables are presented as means ± SDs or medians (interquartile ranges, IQRs). The normality of these data was assessed using the Kolmogorov–Smirnov test. Once the data revealed a non-normal distribution, non-parametric tests were utilized for further analysis [28]. Differences between groups were evaluated using the Mann–Whitney U test for continuous data, while categorical variables such as patient gender and surgical site were expressed as frequencies (percentages) and analyzed using the chi-squared test and Fisher’s exact test as appropriate. The optimal cutoff value for the normal M1-2 distance was determined to be the mean + 2 SDs. Correlations between subgroups were assessed using Spearman’s Rho coefficient, and differences across two or more independent sample subgroups were analyzed with the Kruskal–Wallis test. All statistical procedures were carried out using SPSS version 22.0 (IBM, New York, NY, USA), and a *p* values of less than 0.05 was considered statistically significant.

## 3. Results

### 3.1. Demographics of the Enrolled Cases

This study involved a cohort of 147 individuals: 46 participants in the control group and 101 in the HV group. The average age of the control group was 57.5 years (range, 51.8–65.0), while the HV group averaged 57.0 years (range, 37.0–66.5). HV was clinically diagnosed based on an HVA exceeding 15°, as measured on DP foot X-rays. The final analysis encompassed a total of 207 feet from 147 individuals, comprising 61 feet from the 46 control cases and 146 feet from the 101 HV cases. Demographic comparisons between the control and HV groups showed no significant differences in terms of age, sex, or the affected side of the feet, except for differences in HVA (Table 1).

### 3.2. Correlations between M1-2 Distance and HVA, IMA, and MAAs

**Figure 3:**
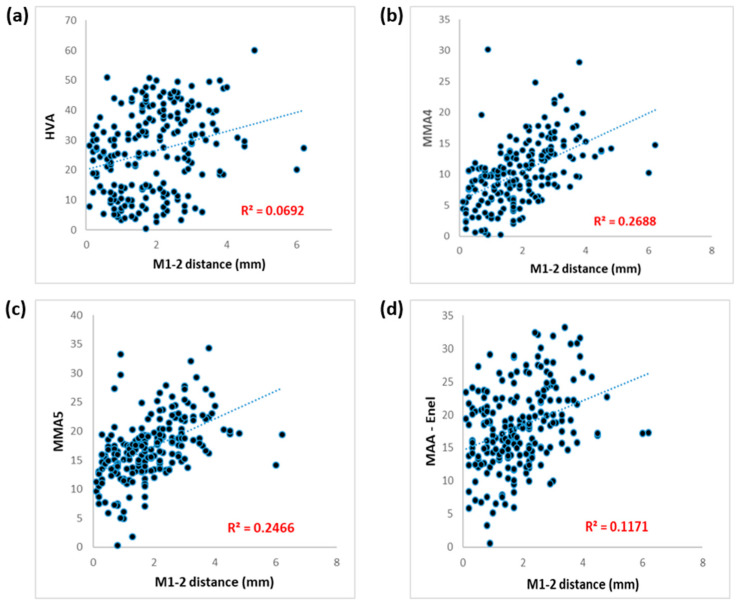
Correlations of M1-2 distance with (**a**) HVA; (**b**) MAA4; (**c**) MAA5; and (**d**) modified Engel’s MAA. M1-2 distance: distance between first and second metatarsal bases; HVA: hallux valgus angle; MAA: metatarsus adductus angle; MAA4: Sgarlato’s MAA; MAA5: modified Sgarlato’s MAA.

There was a significant positive correlation between the M1-2 distance and the HVA, with a Spearman’s Rho coefficient (*r_s_*) of 0.272 (*p* < 0.001). All MAA measurements, including MAA4, MAA5, and modified Engel’s MAA, revealed significant high correlations with M1-2 distance (*p* < 0.001). However, the correlation between M1-2 distance and the IMA was relatively weaker (*r_s_* = 0.171, *p* = 0.014) (Table 2 and Figure 3).

### 3.3. The Difference in M1-2 Distance between Patients with and without MA

To identify participants with MA and analyze its influence on M1-2 distance, cases were stratified into MA(+) and MA(−) groups based on established cutoff values: 14° for MAA4, 20° for MAA5, and 24° for modified Engel’s MAA. Among the cohort of 207 feet, the classifications for MA were as follows: 40 feet (19.3%) were identified as MA(+) using MAA4, 50 feet (24.2%) using MAA5, and 38 feet (18.4%) using modified Engel’s MAA. Notably, the median M1-2 distance was significantly greater in the MA(+) groups compared to the MA(−) groups across all classifications (Table 3).

### 3.4. Comparisons of M1-2 Distance in Different Severities of HV

**Figure 4:**
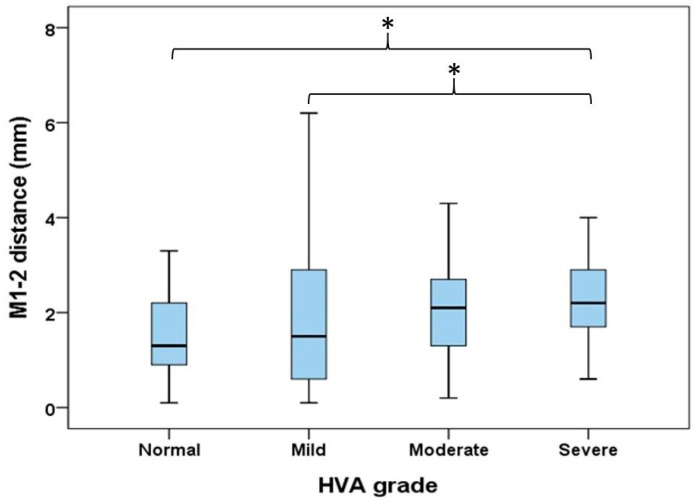
Comparisons of M1-2 distances between different severities of hallux valgus. * *p* < 0.05 between the two subgroups. M1-2 distance: distance between first and second metatarsal bases; HVA: hallux valgus angle.

This study found that all three measurements of MAA increased with the progression of HV and varied significantly across different severities of HV (*p* < 0.05). An increasing trend in the M1-2 distance was also observed with the severity of HV. Specifically, the average M1-2 distances were 1.5 ± 0.8 mm in the normal group, 1.8 ± 1.5 mm in the mild group, 2.0 ± 1.0 mm in the moderate group, and 2.3 ± 0.9 mm in the severe HV group, with these differences reaching statistical significance (*p* < 0.001), as shown in Table 4. Furthermore, post hoc analysis confirmed that these differences in M1-2 distance across subgroups were statistically significant (*p* < 0.05) when comparing the severe HV group to the normal and mild HV groups, as depicted in Figure 4.

### 3.5. The Determination of the Cutoff Value of the M1-2 Distance

**Figure 5:**
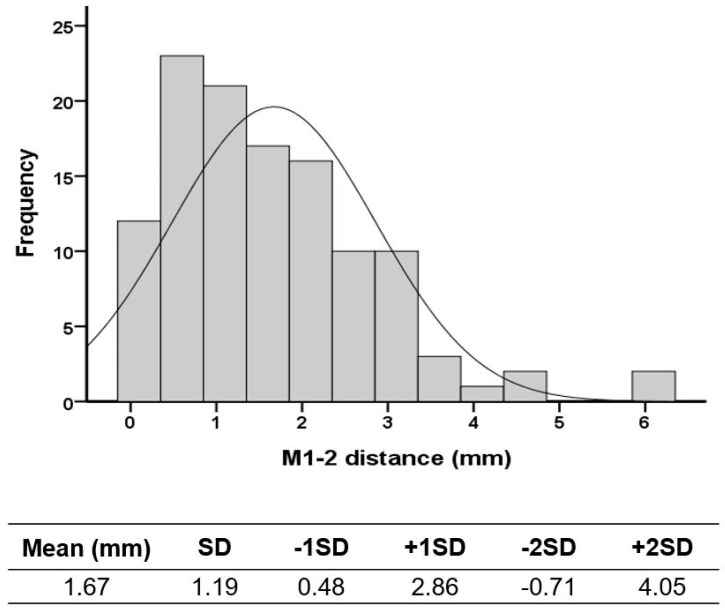
Distribution and cutoff value of M1-2 distance of cohort. M1-2 distance: distance between first and second metatarsal bases; SD: standard deviation.

To reduce the influence of HV severity on the M1-2 distance measurements, the determination of the cutoff value included only the normal and mild HV groups. The analysis included 117 feet in total, comprising 61 normal feet and 56 feet with mild HV. This cutoff value was established at 4.05 mm, calculated as the mean plus two SDs, serving as a threshold to indicate abnormalities in M1-2 distance (Figure 5). This approach ensures that the cutoff value reflects typical conditions without undue bias from severe deformities. In this cohort, 6 out of 207 feet exceeded the cutoff value of 4.05 mm, accounting for 2.9% of the cases. Among these, 66.6% were associated with MA (four out of six cases).

### 3.6. ICC of M1-2 Distance

The ICCs reported for intra-observer and inter-observer reliability in this study were 0.966 and 0.988, respectively (Table 5). These exceptionally high ICC values effectively demonstrate the reliability and repeatability of this measurement.

## 4. Discussion

In this study, 207 feet were analyzed, including 61 normal feet and 146 feet with HV. The results highlighted significant positive correlations between the M1-2 distance and HVA as well as with all three MAA measurements (MAA4, MAA5, and modified Engel’s MAA). Additionally, the median M1-2 distance was notably wider in HV patients diagnosed with MA. A trend was observed where the average M1-2 distance increased with the severity of HV, showing significant differences, particularly between the severe HV group and those with normal or mild severity. This study also established the reliability and repeatability of the M1-2 distance measurements and set a normal cutoff value of 4.05 mm, indicating the threshold for abnormality of the M1-2 distance.

The concept of first-ray instability is characterized by excessive movement of the first metatarsal in both the transverse and sagittal planes. The stability of midfoot joints and the physiological alignment of the hallux are also influenced by foot musculature, and effective collaboration among these muscles helps stabilize the Lisfranc joint in terms of the TMT complex [29]. This multiplanar instability in HV patients can significantly influence various radiographic parameters [20,30]. Prior research has predominantly focused on assessing TMT-1 joint instability in the sagittal plane using tools like the Klaue device [18,22], manual cross-glide tests [31], or the modified Coleman block test [32]. However, studies exploring changes in the transverse plane are limited, largely due to the scarcity of objective evaluation methods. Notably, a study by Young et al. [23] highlighted the usefulness of comparing IMA between weight-bearing and non-weight-bearing anteroposterior foot radiographs as a method for evaluating axial instability. They suggested that an increased IMA during weight-bearing is indicative of greater transverse instability of the TMT-1 joint. This insight highlights the need for the further development and application of methods to assess transverse-plane instability in HV patients with MA.

The IMA, pivotal in the pathology of HV, has been associated with various biomechanical disruptions, including TMT-1 instability during walking [19], and its magnitude often correlates with the severity of HV, as noted by Hardy and Clapham [33]. Contrarily, Aiyer et al. [4] observed that HV patients in the MA group exhibited a smaller IMA compared to those without MA (11.8° vs. 13.8°) in their cohort, suggesting an influence of MA on IMA measurements. This is likely due to the medially deviated alignment of the second metatarsal in MA patients, which leads to an enlarged MAA and a concomitant reduction in the IMA. The disparity in HV severity, based on HVA and IMA, is also evident in the case presented in Figure 2, where a severe HV patient with a large MAA exhibits a small IMA (HVA: 51 degrees, IMA: 13 degrees).

The reduced IMA in the presence of significant MA does not negate the existence of primus metatarsus varus, an important factor in HV pathology. This underestimation of IMA can adversely affect surgical outcomes, potentially leading to higher recurrence rates and the necessity for revision surgeries [4,31]. To address this issue, Avadhoot Kantak advocated for a new radiographic parameter to substitute IMA measurement in MA patients, the metatarsal axis deviation angle (MADA), which shows a high correlation with both the HVA (*r* = 0.6133) and MAA (*r* = 0.5913) [31]. This novel parameter is particularly useful in HV patients with severe MA, as it provides a more accurate depiction of the actual severity of HV, thus aiding in more precise surgical planning. This nuanced understanding emphasizes the importance of comprehensive radiographic evaluation in HV, especially in cases complicated by MA, to better tailor surgical interventions and improve outcomes.

In the context of HV, traditionally, the focus has been on intermetatarsal angular changes; however, this study introduces an innovative perspective by considering transverse hypermobility or instability at the TMT-1 joint, particularly in cases with MA deformity. It is hypothesized that the base of the first metatarsal may shift medially on the TMT-1 articular surface, beyond the angular changes in typical primus metatarsus varus, i.e., IMA, thus exacerbating the distance between the first and second metatarsal bases due to this transverse instability. This research pioneers the definition of the M1-2 distance on standing DP foot radiographs as an indicator of transverse-plane instability at the TMT-1 joint in both normal and HV cohorts. The findings reveal that M1-2 distance is positively correlated with the HVA and the severity of HV, with a specific cutoff value established for normalcy within this cohort.

Ligamentous Lisfranc injuries to the intercuneiform ligament and TMT capsule, which compromise TMT stability, are difficult to detect, especially subtle injuries [34,35]. The M1-2 distance has been considered an effective parameter on conventional radiographs to evaluate TMT instability in ligamentous Lisfranc injuries [36]. Lisfranc injury should be suspected if the M1-2 distance is greater than 4 mm in non-weight-bearing radiographs, and greater than 5 mm under weight-bearing conditions [37]. Similarly, transverse TMT instability in HV should also increase the M1-2 distance. In this cohort, the cutoff value, defined as the mean plus 2 SDs, was determined to be 4.05 mm on weight-bearing radiographs. Based on the definition of the upper limit value (mean + 2 SDs), the M1-2 distance exceeded this value in only 2.5% of HV cases. This indicates that significant transverse instability of the TMT-1 joint should be considered if the measured M1-2 distance surpasses the cutoff value. Notably, the M1-2 distance in severe HV cases significantly differs from that observed in normal and mild HV cases, suggesting the presence of transverse TMT instability in severe HV. Additionally, the M1-2 distance is significantly greater in HV feet with MA compared to those without, suggesting that transverse instability may be a contributing factor to MA in HV patients. Therefore, these insights suggest that addressing the multiplane instability of the first ray, particularly through TMT-1 reorientation arthrodesis, may be a viable surgical intervention for severe HV patients with significant MA, offering a targeted approach to managing complex foot deformities. This approach highlights the importance of a comprehensive assessment of both angular and translational deformities in the surgical planning and treatment of HV, particularly when complicated by a large MAA.

We acknowledge several limitations of this study. Its retrospective design may introduce potential for selection bias and confounding factors that are unaccounted for. Additionally, this study had a relatively small sample size and was conducted at a single institution. To address these issues and verify the findings, future research should employ a prospective design, ideally involving a larger, more diverse sample from multiple centers. Another limitation is that this study was purely radiographic, and did not include clinical outcome measures or physical examinations. Future studies are needed to explore how the M1-2 distance in HV patients with MA influences clinical outcomes, and to investigate the association between M1-2 distance and objective validated measurement techniques for transverse TMT-1 instability. These studies will provide more comprehensive insights into the practical implications of the observed radiographic changes.

## 5. Conclusions

In this study, the M1-2 distance was highlighted as a crucial radiographic marker in HV patients with MA deformity. As the MAA increased along with HV severity, the M1-2 distance was found to significantly expand. Notably, significant differences in the M1-2 distance between groups with and without MA were observed. Although the increasing M1-2 distance implies potential hypermobility of the TMT-1 joint on the axial plane of weight-bearing foot radiographs, the direct correlation between M1-2 widening and transverse instability should be further elucidated in future research. However, the potential transverse instability of the TMT-1 joint should be considered if the measured M1-2 distance exceeds 4.05 mm. These deeper insights into MA in HV are instrumental for clinicians, facilitating more optimal surgical decisions, particularly for HV cases with severe MA where potential TMT-1 instability is a concern.

## Figures and Tables

**Table 1:** Demographic characteristics of enrolled cases.

	Control	HV	*p* Value
Patients (N)	46	101	
Age	57.50 (51.8–65.0)	57.00 (37.0–66.5)	0.887
Sex			0.203
Female	32 (69.6%)	80 (79.2%)	
Male	14 (30.4%)	21 (20.8%)	
Feet (N)	61	146	
Side			0.693
Right	27 (44.3%)	69 (47.3%)	
Left	34 (55.7%)	77 (52.7%)	
HVA (degrees)			<0.001 **
≤15	61 (100.0%)	0 (0.0%)	
>15	0 (0.0%)	146 (100.0%)	

Data presentation: frequency (percentage), median (IQR). Chi-squared test or Mann–Whitney U test. ** *p* < 0.01. HV: hallux valgus; HVA: hallux valgus angle.

**Table 2:** Correlation of M1-2 distance with HVA, IMA, and MAAs.

	M1-2 Distance	
	*r_s_*	*p* Value
HVA	0.272	<0.001 **
IMA	0.171	0.014 *
MAA4	0.574	<0.001 **
MAA5	0.569	<0.001 **
Modified Engel’s MAA	0.341	<0.001 **

Spearman’s Rho Coefficient. * *p* < 0.05, ** *p* < 0.01. M1-2 distance: the distance between the first and second metatarsal bases; HVA: hallux valgus angle; IMA: intermetatarsal angle; MAA: metatarsus adductus angle; MAA4: Sgarlato’s MAA; MAA5: modified Sgarlato’s MAA.

**Table 3:** Comparisons of M1-2 distance in MA(+) and MA(−) groups.

	N (%)	M1-2 Distance (mm)	*p* Value
MAA4 (deg.)			<0.001 **
≤14	167 (80.7%)	1.6 (0.8–2.2)	
>14	40 (19.3%)	2.9 (2.3–3.4)	
MAA5 (deg.)			<0.001 **
≤20	157 (75.8%)	1.5 (0.8–2.2)	
>20	50 (24.2%)	2.8 (2.2–3.3)	
Modified Engel’s MMA (deg.)			<0.001 **
≤24	169 (81.6%)	1.6 (0.8–2.3)	
>24	38 (18.4%)	2.7 (1.8–3.0)	

Data presentation: frequency (percentage), median (IQR). Mann–Whitney U test. ** *p* < 0.01. M1-2 distance: distance between first and second metatarsal bases; MA: metatarsus adductus; MAA4: Sgarlato’s MAA; MAA5: modified Sgarlato’s MAA.

**Table 4:** The differences in M1-2 distance and MAAs with various HVA grades.

	Normal	Mild	Moderate	Severe	*p* Value
M1-2 distance (mm)	1.5 ± 0.8	1.8 ± 1.5	2.0 ± 1.0	2.3 ± 0.9	0.001 **
MAA4 (deg.)	8.81 ± 5.19	10.11 ± 4.27	10.80 ± 4.31	11.67 ± 6.46	0.026 *
MAA5 (deg.)	15.45 ± 6.25	16.77 ± 4.09	17.75 ± 5.15	18.92 ± 5.70	0.028 *
Modified Engel’s MAA (deg.)	14.92 ± 5.71	18.27 ± 5.26	19.93 ± 5.71	20.74 ± 6.77	<0.001 **

Data presentation: mean ± SD. Kruskal–Wallis test. * *p* < 0.05, ** *p* < 0.01. M1-2 distance: distance between first and second metatarsal bases; MAA: metatarsus adductus angle; HVA: hallux valgus angle; MAA4: Sgarlato’s MAA; MAA5: modified Sgarlato’s MAA.

**Table 5:** Interclass correlation coefficient (ICC) of M1-2 distance.

	ICC	95% CI	*p* Value
		Lower–Upper	
Intra-observer	0.966	(0.937–0.982)	<0.001 **
Inter-observer	0.988	(0.977–0.994)	<0.001 **

** *p* < 0.01. M1-2 distance: distance between first and second metatarsal bases; ICC: interclass correlation coefficient; CI: confidence interval.

## Data Availability

The datasets used and analyzed during the current study are available from the corresponding author upon reasonable request.
